# Bulk Genotyping of Biopsies Can Create Spurious Evidence for Hetereogeneity in Mutation Content

**DOI:** 10.1371/journal.pcbi.1004413

**Published:** 2016-04-22

**Authors:** Rumen Kostadinov, Carlo C. Maley, Mary K. Kuhner

**Affiliations:** 1 Pediatric Oncology, Johns Hopkins School of Medicine, Baltimore, Maryland, United States of America; 2 Center for Evolution and Cancer, University of California, San Francisco, San Francisco, California, United States of America; 3 Department of Genome Sciences, University of Washington, Seattle, Washington, United States of America; University of Calgary Cumming School of Medicine, CANADA

## Abstract

When multiple samples are taken from the neoplastic tissues of a single patient, it is natural to compare their mutation content. This is often done by bulk genotyping of whole biopsies, but the chance that a mutation will be detected in bulk genotyping depends on its local frequency in the sample. When the underlying mutation count per cell is equal, homogenous biopsies will have more high-frequency mutations, and thus more detectable mutations, than heterogeneous ones. Using simulations, we show that bulk genotyping of data simulated under a neutral model of somatic evolution generates strong spurious evidence for non-neutrality, because the pattern of tissue growth systematically generates differences in biopsy heterogeneity. Any experiment which compares mutation content across bulk-genotyped biopsies may therefore suggest mutation rate or selection intensity variation even when these forces are absent. We discuss computational and experimental approaches for resolving this problem.

## Author Summary

Researchers who take multiple samples from a cancer or pre-cancer tissue and find that some samples show far more mutations than others are likely to conclude that the high-mutation samples reflect cells with an abnormal mutation or growth rate. We considered the common practice of testing a bulk sample for mutations, which finds only mutations that are common within the sample. Our computer simulations show that even when all cells have identical mutation and growth rates, testing bulk samples frequently leads to spurious detection of rate differences. This can lead to false conclusions about the causes and progress of cancer. We discuss possible solutions involving either genetic testing of single cells or the use of computer algorithms to detect rare mutations within a sample.

## Introduction

The somatic genotypes of cancerous and pre-cancerous tissues are frequently assayed by taking biopsies containing a substantial number of cells and genotyping each biopsy as a whole (via SNP chip, exome or genome sequencing, or other techniques). For example, in a study of Barrett’s esophagus genotypes were derived from biopsies containing approximately one million epithelial cells (e.g. [1]). We will refer to this type of data collection as bulk-biopsy genotyping. It is generally much less expensive and technically difficult than single-cell genotyping. In this study we examine the use of multiple biopsies from a single tissue or tumor. It is tempting to think that differences in mutation content observed in bulk-biopsy genotyping reflect underlying differences in the number of mutations per cell, which could be informative about the spatial or temporal evolution of the tissue. But is this really true?

The roles of mutations in cancer have often been assessed by analyzing single tumor samples or paired tumor/normal samples from many patients. However, recently it has been recognized that multiple samples from a single patient, separated in time or space, offer additional information. Spatial heterogeneity of clones implies that sampling a single region of a neoplasm may not be representative of the entire neoplasm. The force of natural selection may vary in different parts of a tissue (edge versus center, primary tumor versus metastasis) or over time (early versus late progression, before versus during or after chemotherapy). Multiple samples from a single individual also offer the possibility of phylogenetic analysis to infer relationships among different lineages and reconstruct past events in the history of the tissue. Table 1 shows a sampling of recent studies in which multiple cancer samples per patient were obtained, and phylogenetic methods were either used or could have been used. These studies considered both spatial separation–different parts of a tumor or neoplasm, a tumor and its metastases– and temporal separation– samples taken at different times, such as early and late in progression to cancer, or before and after chemotherapy. They show the potential power of the multiple-sample approach, which we expect will become increasingly important as genotyping costs decrease.

**Table 1:** Studies with multiple-sample data suitable for phylogenetic analysis. WGS, whole genome sequencing.

Phylogenetic analysis used			
Cases	Condition	Method	Reference
22	CLL	Targeted resequencing	[2]
1	Barrett’s esophagus	SNP arrays	[3]
2	Breast cancer	Single-cell WGS	[4]
30	ALL	FISH	[5]
4	Renal-cell carcinoma	WGS	[6]
21	Breast cancer	WGS and SNP arrays	[7]
1, 2	DS-ALL and ALL	WGS, single cell targeted mutational profiling	[8]
2	Breast cancer	Single cell WGS	[9]
13	Barrett’s esophagus	SNP arrays	[1]
23	Glioma	Exome sequencing	[10]
15	Colorectal adenoma and carcinoma	Multiple methods	[11]
Phylogenetic analysis feasible			
Cases	Condition	Method	Reference
7	Pancreatic cancer	Exome sequencing and SNP arrays	[12]
21	ALL	SNP arrays	[13]
7	MDS and sAML	WGS	[14]
8	Acute myeloid leukemia	WGS	[15]
258	Barrett’s esophagus	SNP arrays	[16]

Existing methods do not have the resolution to detect all variants present in a million-cell sample. Variants present in just a few cells will go undetected. Peiffer et al. [17] found minimum frequencies of 33% to 50% for reliable detection of copy-number variants in heterogeneous tumor data using a SNP array. Deep sequencing can detect single nucleotide variants in cancer samples at lower frequencies, down to 1% [18] for 40x sequencing, but the threshhold for reliable detection is much higher: even for an average of 2000x sequencing, single nucleotide variants were reproducibly detected only at >15% allele frequency and indels at >5% allele frequency [19]. Even when low-frequency variants are detected, they are often disregarded as they are difficult to quantify and assign to haplotypes. Thus, the result of bulk-biopsy genotyping is generally a survey of locally high-frequency variants only.

In a well-mixed tissue such as blood, variants which are at high frequency in one sample will generally be at high frequency in all samples. In such tissues, bulk genotyping will miss low-frequency variants, and will thus be biased toward detecting older rather than younger mutations. However, this bias will affect all samples equally and will not tend to produce spurious evidence of non-neutrality.

However, solid tissues are not well-mixed. We will consider the behavior of bulk-biopsy genotyping in a simulated tissue similar to Barrett’s esophagus (BE): a sheet of tissue rolled into a cylinder, with very limited mobility of cell lineages except during initial development. While our simulations are inspired by BE, our conclusions should apply directly to neoplasms in two-dimensional epithelial sheets such as colon, skin, bladder and lung, and conceptually similar effects are also likely in three-dimensional tumors. The key factor is growth with limited mixing.

An increasingly common objective in taking multiple biopsies from a neoplastic tissues is to look for evidence of natural selection or heightened mutation acting on specific clones. This is distinct from standard methods of detecting selection or enhanced mutation via comparison of single samples from many different tumors. A straightforward statistical approach to detecting perturbing forces from multi-sample data would be to infer the evolutionary tree connecting samples from the same individual, and test if that tree conforms to a molecular clock. We simulate this experiment on data which do have a molecular clock, and show that bulk-biopsy genotyping very often leads to the spurious rejection of the clock, and thus to a conclusion of non-neutrality, even when the underlying data are completely neutral.

We emphasize that the bias we observe is not specific to the use of a phylogeny-based molecular clock test, but will influence any formal or informal comparison of apparent mutation content differences among biopsies. For example, if researchers use bulk genotyping to identify a biopsy with an unusually high number of mutations, and conclude that the highly mutant biopsy represents a genetically unstable lineage, they are implicitly assuming that bulk data have a molecular clock in the absence of perturbing forces. As we will show, this is not the case.

## Methods

### Model of the BE segment

We model the BE segment as a 300 x 300 grid of crypts rolled into a cylinder, approximating the size of a typical BE segment. We treat a crypt as the basic replicative unit, since genetic drift is expected to rapidly homogenize the genotype within each crypt. Crypts have a birth rate representing crypt fission. According to Totafurno’s model of the crypt cycle, when stem cells double in number crypt fission is triggered, which results in halving the doubled stem cell population into two new daughter crypts [20]. In this study, we model the crypt fission cycle by allowing a crypt to either eliminate a neighboring crypt or fill in a space lacking crypts. Crypts also have a death rate, a dead crypt leaving an empty space.

The BE segment is thought to expand from the gastro-esophageal junction. An alternative possibility is that cells gain a BE phenotype and spread clonally from squamous duct glands that are situated throughout the esophagus [21]. In both scenarios, BE segments must be rapidly established, since BE segments have not been endoscopically observed in the process of expanding. However, when we simulated the esophagus beginning from a uniform field of non-mutant cells no tree structure arose even after many simulated decades. We do not show results for this case as it is trivially predictable from coalescent theory: our simulations cover approximately 50 generations, and a thoroughly mixed population of size 90,000 will have an average of 1765 distinct ancestors 50 generations ago and will thus appear as approximately 1765 unrelated patches. A less thoroughly mixed sheet of cells will be even patchier. This is not consistent with actual BE data [1] which show mutations shared among biopsies. It is possible that BE arises *in situ* and is then “overwritten” by an early selective sweep; but if so, this seems little different from the BE segment itself arising by growth from one or a few ancestral crypts. We therefore model the establishment of BE as an expansion from the gastro-esophageal junction.

### Simulations

To simulate BE data we used the agent-based forward simulator of [22]. While this simulator provides for loci whose mutant alleles modify the growth or mutation rates, in the majority of experiments presented here we used a purely neutral model. We simulated 1000 neutral loci for phylogeny inference. Mutations were scored as number of changes from ancestral state; there was no back mutation. We considered neutral mutation rates per locus per crypt per year (*μ*) of 0.001 and 0.002. Data with the lower rate are fairly sparse, while data with the higher rate are highly polymorphic. We set the probability that a dividing crypt could displace a neighbor at 1. The crypt birth rate was 0.02 and death rate 0.001. We also did simulations with 100 neutral loci and mutation rates of 0.001, 0.002, and 0.004, presented in Supporting Information.

For illustrative purposes we also did a small number of simulations with five potentially selected loci, each having a mutation rate of 10^−7^ per locus per crypt per year and a twofold selective advantage for the mutant type over the wild type.

The mutation rates given here do not correspond directly to per-cell mutation rates since, when a mutation arises in a crypt, it may be lost rather than fixed. The per-cell mutation rate would be higher by a factor of the mean number of stem cells in the crypt. In any case our mutation rates are chosen in order to give ample mutations for phylogenetic analysis with a limited number of loci. Real data would have fewer mutations per locus, but far more loci. We do not expect this to substantially change the results.

Numerical estimates of BE crypt birth and death rates are not available. Our chosen numbers, which were roughly inspired by values measured for human colon crypts [23], produce an initial spread which is slower than in BE, and a subsequent steady state which probably has faster turnover. However, this is conservative for our conclusions: a faster initial spread and slower subsequent turnover with the same expected amount of mutation would show even greater distortion of the molecular clock. We believe that the details of our parameter choice will not affect our qualitative conclusions as long as the pattern of rapid spread followed by slow turnover is conserved.

We started with a single randomly placed crypt and simulated 20 years of growth. This was generally enough to allow crypts to fill the lower esophagus. A small proportion of simulations resulted in the death of the nascent Barrett’s epithelium; these were discarded. We then randomly chose 10 biopsies which were squares of 10x10 crypts, constrained not to overlap. Rarely, a biopsy was found to contain no live crypts; in such cases the entire simulation was discarded. Additional simulations were run to replace discarded simulations.

These simulation conditions imply a molecular clock, as the mutation rate is the same in all crypts. We tested for presence of a clock in single crypt samples and in biopsies of different sizes using PAUP* 4.0 [24]. For analytic purposes we treated all loci with one or more mutations as one state, and loci with zero mutations as another state. This corresponds to the presence/absence scoring typically used for BE data. To enable use of available phylogenetic software, these states were coded as purine and pyrimidine ambiguity codons. We tested both estimation of the state frequencies from the data using the EMPIRICAL algorithm in PAUP* (results shown in paper), and setting the frequencies equal (results shown in Supporting Information). We performed maximum-likelihood analyses of the recoded data with and without the clock constraint, and assessed the difference in log-likelihoods using a likelihood ratio test [25, 26] with a 5% significance cutoff. When multiple tied trees were produced, we used the first listed tree for analysis.

This use of the likelihood ratio test can be criticized as it assumes that the clocklike and non-clocklike best trees had the same topology [26], which was not always the case. We applied the test to all pairs of trees, even those differing in topology. Our argument is that when the topological difference is trivial (rearrangement across branches of near-zero length) the result of the test will be almost exactly the same as it would for identical topologies; and when the topological difference is non-trivial rejection of the clock is justified even though an exact statistical test is not available.

To measure the influence of biopsy size on detection of rate heterogeneity, we subsampled our biopsies. That is, to produce a 4x4 biopsy we took a 4x4 subsample from the original 10x10 biopsy. To avoid dead crypts, we examined subsamples in turn starting in the upper left and chose the first one in which at least 1 live crypt was found.

To measure the influence of detection threshholds, we used the same sets of simulated biopsies, but varied the cutoff used to establish the biopsy “genotype.” For example, when the cutoff was 30%, we scored a mutation as present if it appeared in 30% or more of the sampled living crypts from the biopsy, and absent otherwise.

In the simulations with 100 loci and *μ* = 0.001, which had the smallest amount of information per phylogeny, a few cases with large biopsies and stringent cutoffs could not be run. Stringent cutoffs can generate biopsies with no detectable mutations, and having too many such biopsies in a single tree causes failure of the phylogeny analysis. Such runs were discarded. No more than 15/500 runs failed for any combination of conditions; the number of failed runs for each condition are given in the legends to S5 and S6 Tables.

Our simulated data is archived on Dryad at http://dx.doi.org/10.5061/dryad.hf93c.

## Results

Our simulations were inspired by Barrett’s esophagus (BE), a neoplastic condition in which the lower esophagus is colonized by a tissue organized into crypts. We treat crypts as the fundamental unit of our simulation, and assume that all spread of genotypes results from reproduction (fission) of crypts which either replace their neighbors or spread into unoccupied areas. The details of the simulator are described in [22].

At the beginning of the simulation each crypt began with an identical genome of 100 or 1000 loci. Mutations in these loci were selectively neutral: they were used solely to infer the relationships among biopsies.

The first striking effect of bulk sampling was seen when the simulation was seeded with a completely filled grid of crypts. At the end of the simulation the tissue consisted of tiny patches of related crypts, each patch unrelated to its neighbors. This reflects the very low gene flow in a static crypt-organized tissue without natural selection. In a tissue of this kind, bulk genotyping would lead to the incorrect conclusion that there are few or no mutations present.

Bulk biopsy sampling of actual BE segments shows abundant mutations [1]. We therefore considered a theory of BE origin in which it spreads from a few crypts. We represented this by seeding the simulation with a single randomly placed crypt. Biopsies sampled from such a tissue did contain genetic variants detectable with bulk genotyping, consistent with actual BE data.

**Fig 1:**
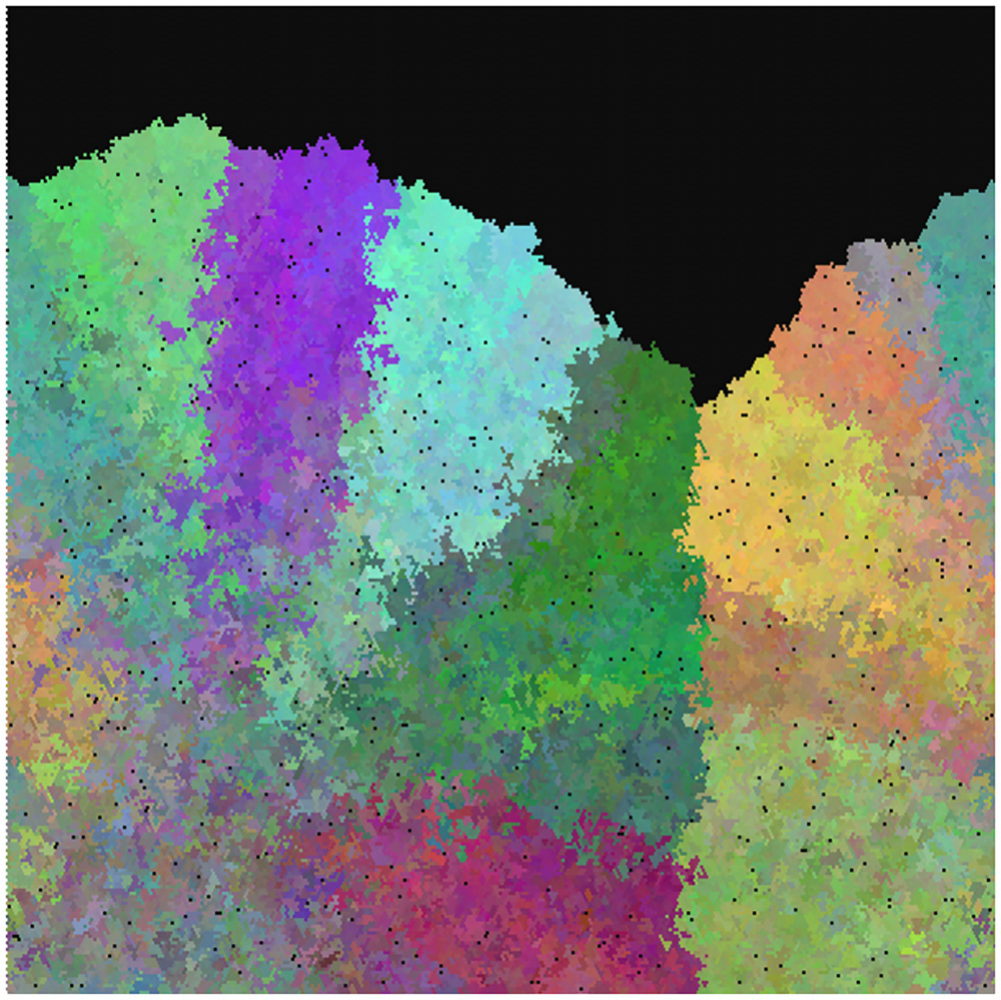
Simulated BE segment. Black regions represent not-yet-colonized areas of the esophagus, and small black dots represent crypts which have died and not yet been replaced. Colors indicate the first three principal components of a PCA analysis of crypt genotypes at 1000 neutral loci, essentially labeling distinct clones or cell lineages. Initial seeding of the esophagus was along the lower border of the figure. The left and right sides of the image wrap around.

The spatial distribution of mutations in real BE segments is poorly known, as normally only a few biopsies are analyzed per individual. In our simulations we could readily examine the entire pattern, as well as taking simulated biopsies. The simulated BE segments developed a strongly sectored pattern, with small diverse patches of cells near the original seeding area, and larger, more homogeneous patches far from it. Sharp borders between genetically distinct lineages were seen; these borders ran vertically along the simulated esophagus, roughly parallel to the direction of tissue growth. A typical example, captured partway through colonization of the simulated esophagus, is shown in Fig 1.

These patterns reflect the effect of “gene surfing” [27]. Gene surfing is a phenomenon in population genetics, observed when a population is rapidly expanding into a new geographical region but the mobility of individuals is limited. Colonization is therefore driven by a few individuals on the leading edge of the population, and their genotypes will be disproportionately represented in the newly colonized area. Patterns visually similar to our simulations can be seen when two different strains of bacteria are mixed and seeded onto a plate: sectors of pure strains are generated by replication of the few individuals on the colony edge [28] even in the absence of any selective advantage.

Our simulations, seeded with a single crypt, thus produced data that were broadly consistent with observations of actual BE segments. We next asked whether biopsies sampled from these purely neutral simulations would pass tests for neutrality. Based on ten biopsy samples from each of 100 simulated BE segments, we inferred phylogenetic trees and tested whether those trees rejected the molecular clock at the 5% level. We considered biopsies of sizes from 1 (a single crypt) to 10x10 (100 crypts). For biopsies of size greater than 1, we also considered detection cutoffs from 10% (mutations present in 10% or more of crypts were scored) to 100% (only mutations present in all crypts were scored).

**Fig 2:**
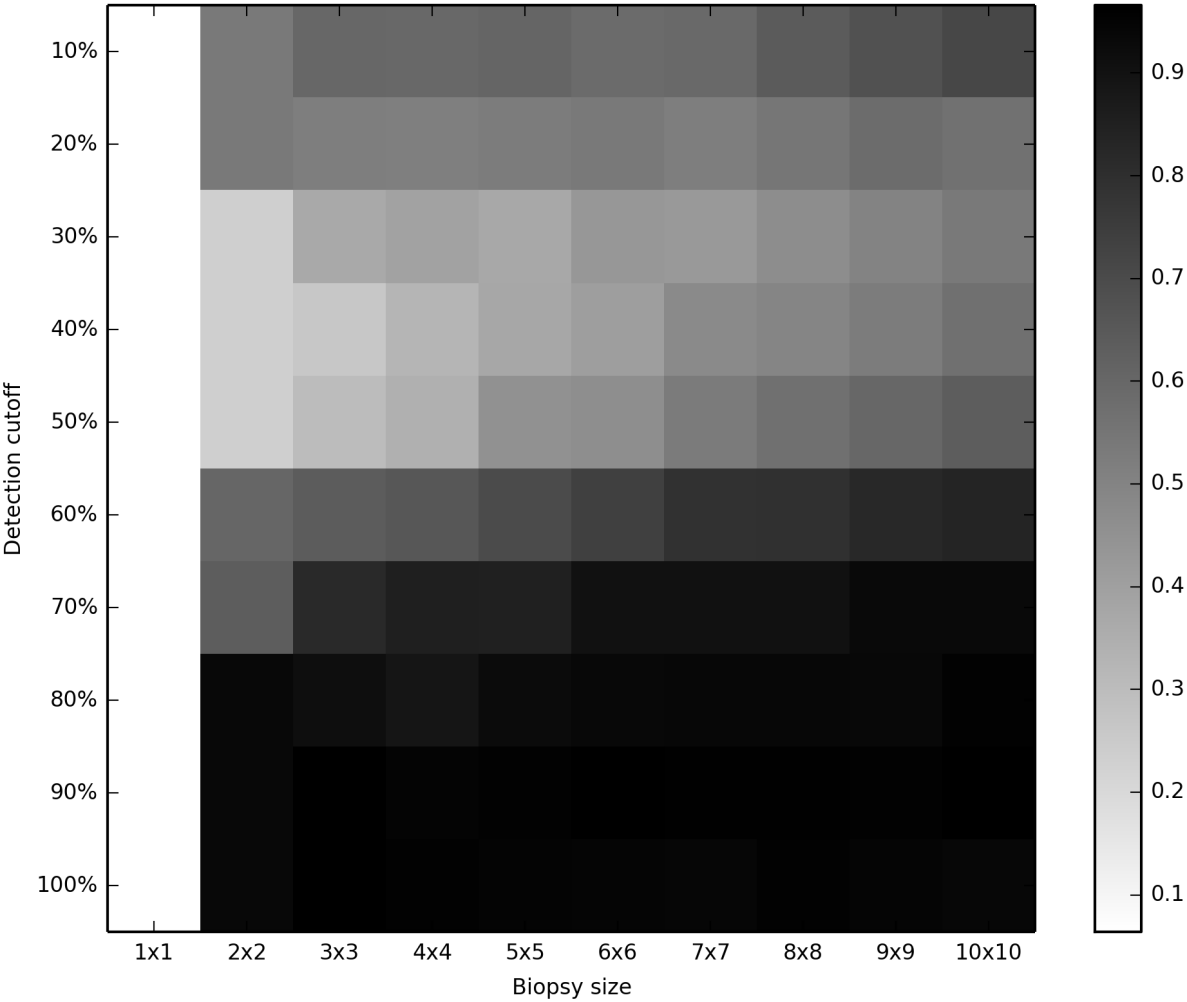
Rejection of the clock with 1000 neutral loci, *μ* = 0.001. Heat-map showing the percentage of cases rejecting the molecular clock at the 5% level; lighter colors indicate lower rejection of the clock.

**Fig 3:**
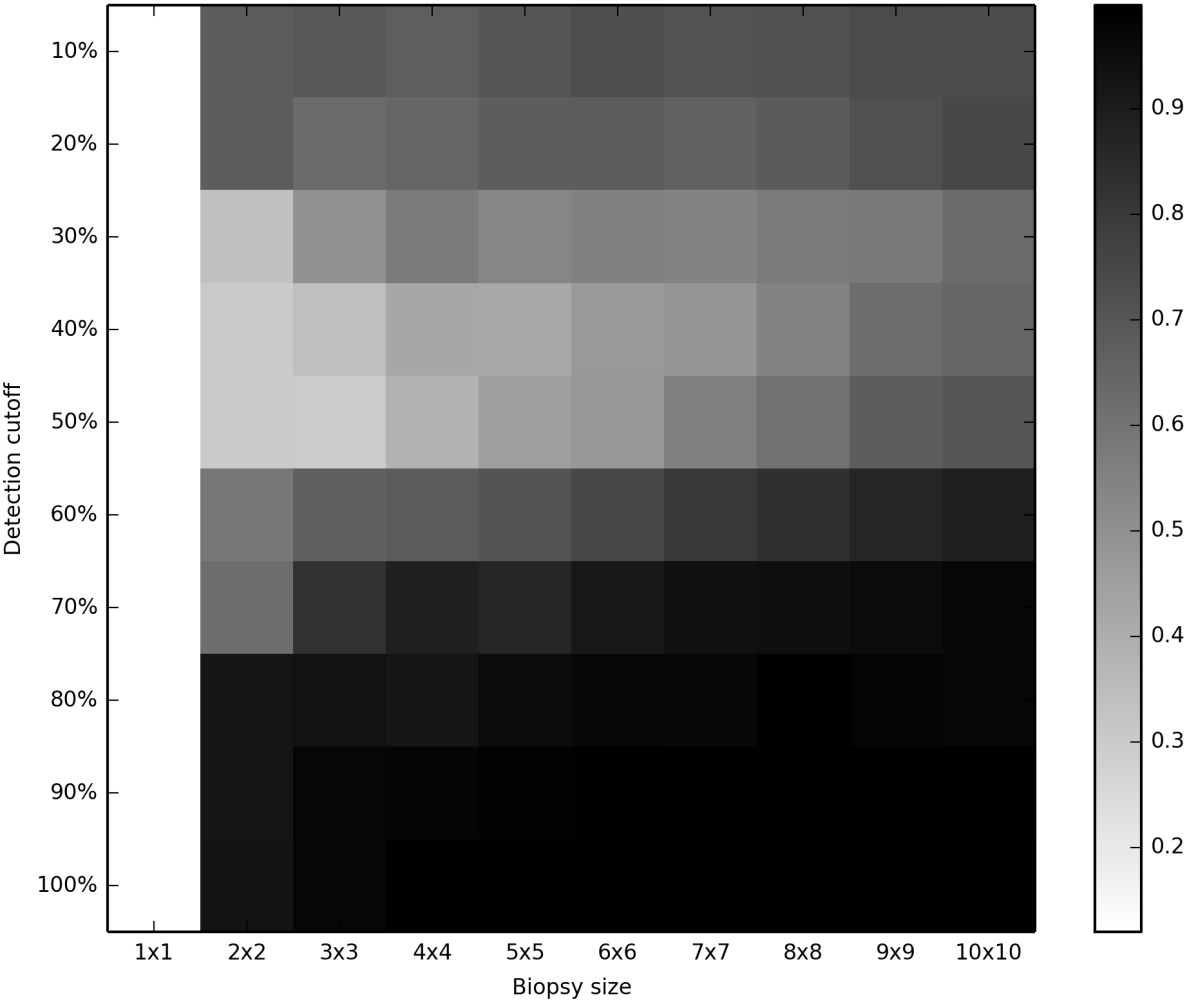
Rejection of the clock with 1000 neutral loci, *μ* = 0.002. Heat-map showing the percentage of cases rejecting the molecular clock at the 5% level; lighter colors indicate lower rejection of the clock.

If biopsy sampling provided a phylogenetically unbiased sample of mutations occuring in our data, we would expect to see a molecular clock in our inferred trees with any size of biopsy. The proportion of inferences (out of 500) rejecting the molecular clock are shown in Figs 2 and 3 and are presented in table form in S1 and S2 Tables. In these figures, the white color seen at the left-hand edge (single-crypt samples) represents an acceptable clock rejection rate of 5%. (Note that detection cutoff does not affect the results from single crypts, and thus all of the left-hand results represent the same analyses.) All larger biopsy sizes, even 2x2 biopsies with only 4 crypts, rejected the clock at high rates for all conditions studied.

The choice of cutoff had a noticable impact on clock rejection. Cutoffs in the 30%-50% range were better than higher or lower cutoffs; the larger the biopsy, the lower the optimal cutoff. However, no cutoff tested restored the clocklike nature of the underlying data.

Superficially satisfactory results can be obtained by using only 100 neutral loci and inferring frequencies of the mutant and non-mutant states (S3 Fig). However, this apparent improvement merely represents lack of statistical power to detect clock violations, as seen by the dramatic worsening of results with 1000 neutral loci and the same model (Fig 2).

Use of equal frequencies of mutant versus non-mutant states produces higher clock rejection: results are shown for completeness in S1 and S2 Figs for 1000 neutral loci and S4, S5 and S6 Figs for 100 loci.

**Fig 4:**
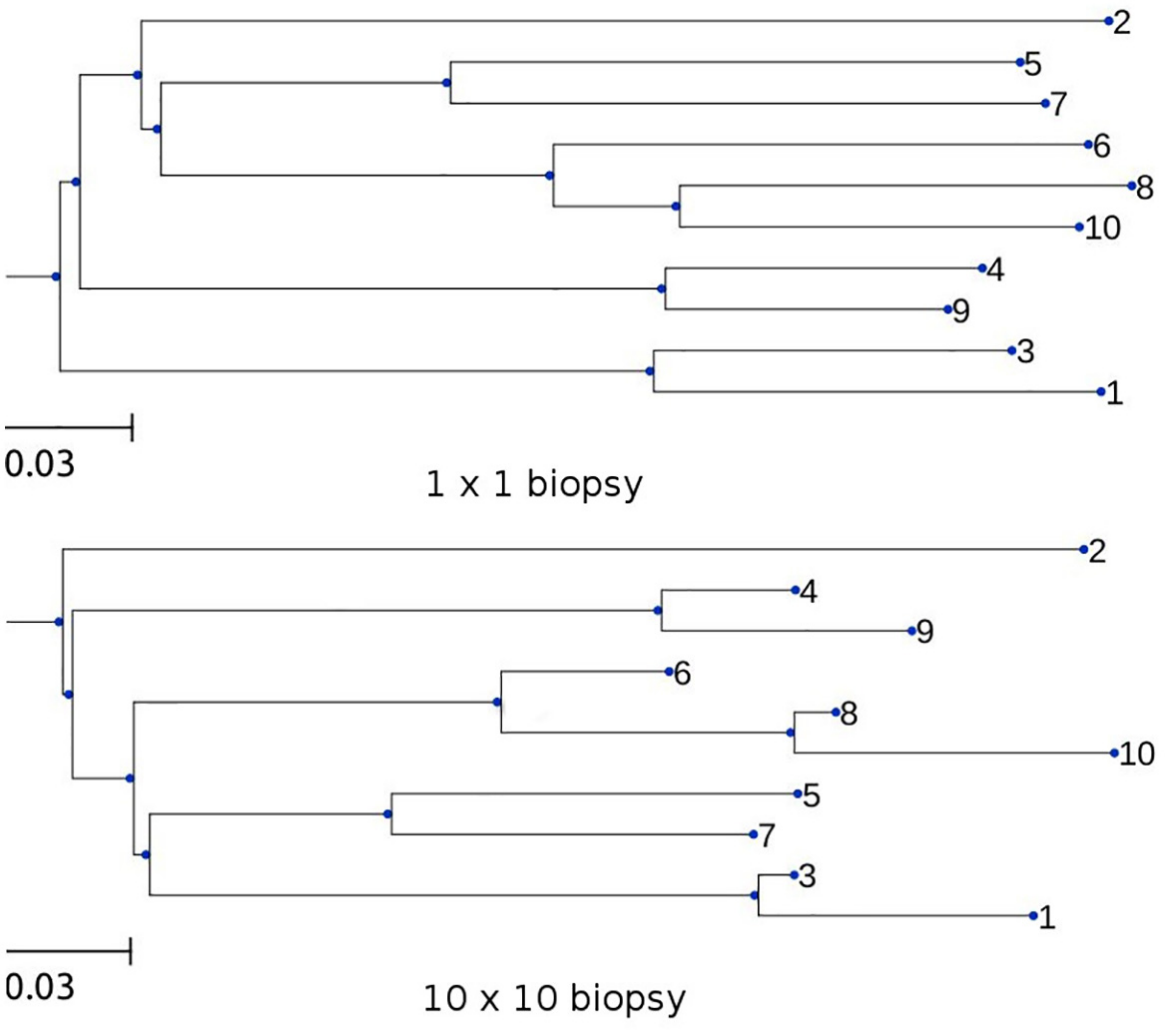
Single-crypt and bulk-biopsy inferred trees. Trees were inferred, without assuming a clock, from the simulation of S6 Table. Upper panel, sample of size 1 crypt. Lower panel, bulk sample of size 10x10 crypts using a 40% cutoff.

We show a randomly selected pair of inferred trees from the simulation of S6 Table in Fig 4. The topologies of the two trees differed in ordering of the short bottommost branches. Larger discrepancies were seen in the branch lengths. The single-crypt tree (A) showed some heterogeneity of branch lengths, but it was well within the expected range for a data set of this size, and the clock was not rejected. The 10x10 crypt tree (B) was much more distorted, and rejected the molecular clock. It would be tempting to conclude that biopsy 10, in particular, had a higher mutation rate than biopsy 8; yet they arose from a simulation with perfectly equal rates.

## Discussion

We have presented our results in terms of rejection of the molecular clock in a formal test. However, their significance is not limited to such tests. When we first examined bulk-genotyped BE data for multiple biopsies per patient we saw a striking difference in the mutation content of different biopsies. It was natural to read this as a difference in the underlying mutation rate. After further thought we realized that it could also reflect a difference in the growth rate, since rapidly growing cells will form more uniform samples and therefore appear to have more mutations. Only after performing simulations did we discover that hetereogeneity in apparent mutation content is a general feature of this type of data and should be expected even when neither mutation rate nor growth rate varies. Comparison of Figs 2 and S5 shows that the more informative the data, the stronger this tendency to reject the clock. The clock test formalizes a scientist’s intuition, but both the test and the intuition are liable to error in this case.

We stress that our findings do not challenge the important role non-neutral processes play in the development of cancer. Instead, they warn us against drawing conclusions about non-neutrality that cannot be supported.

Two factors combine to produce this spurious evidence for non-neutrality. Gene surfing causes biopsies taken near the origin of the growing population to be much more heterogeneous than those taken far from the origin (see Fig 1). Bulk-biopsy sampling then translates this difference in diversity into a difference in detectable mutation content: a homogeneous cell sample will have more high-frequency mutations than a heterogeneous one, and bulk sampling detects only high-frequency mutations.

This is most easily understood by considering common ancestry. Consider, as an example, a detection cutoff of 50%. Mutations which reach this cutoff must exist in 50% or more of the cells in the biopsy, and thus, barring convergent evolution, must be inherited from a common ancestor of 50% of the cells. If this common ancestor existed early in the development of the tissue, it likely had relatively few mutations, so few mutations will be shared by its descendants. If it existed more recently, it likely had more mutations (since mutations accumulate over time) and its descendants will have more shared mutations. Cells from a biopsy whose common ancestor is ancient will, individually, have just as many mutations, but a much larger proportion will be at low frequency in the biopsy. Such mutations are difficult to detect with bulk genotyping.

We did not model complicating factors in analysis of bulk data such as differences in ploidy among lineages or typing errors. However, when a bias is present in analysis of clean, error-free genotypic data, there is no reason to believe that better results would come from dirtier data.

Regrettably, no tested frequency cutoff rule was successful in resolving this problem. We suggest three possible approaches.

(1) Examine single-cell or single-crypt samples in preference to bulk genotyping. Single-crypt samples from our simulation saw a molecular clock even though all larger samples did not. Small sample genotyping (single crypts or cells) is technically challenging but possible in some systems [9, 29]. This approach completely avoids the hazards of bulk genotyping. However, a pure small-sample genotyping strategy may be inefficient, as little information is gained about mutation frequency unless a large number of small samples are genotyped. A single small-sample genotype is probably less useful than a bulk genotype for detecting biologically critical mutations such as putative “drivers” as it will also contain many biologically irrelevant private polymorphisms.
(2) Combine small-sample and bulk genotyping. For example, a sample could consist of a bulk genotype of the whole biopsy and individual genotypes of a few cells or crypts from it, as done for example by [11]. The single crypts provide information about the mutation content of individual crypts, while the bulk samples provide frequency information [30]. A biopsy in which the individual crypts show far more mutations than the bulk sample is revealed to be heterogeneous, and frequencies inferred from it can be viewed in this light.
(3) Determine genetic diversity within a biopsy by computational means. Considerable recent work has focused on determining the number and relative frequencies of different lineages in a biopsy [31–33]. The problem is extremely challenging because different cells may vary in underlying ploidy as well as in mutation content. Progress in this area may require both computational improvements in deconvolution algorithms and experimental improvements in data collection.

Once subclones within a biopsy have been detected via approaches (2) or (3), this information needs to be incorporated into the analysis. For analytic methods involving phylogenetics, mixed samples are particularly challenging because when two variants are found at similar frequency in a sample, there is no easy way to determine whether they represent one lineage with two mutations or two lineages with one mutation each [33]. In principle it would be possible for a statistical analysis to sum over these possibilities using an approach analogous to that of [34]. The high computational burden of this approach will have to be compared with the experimental burden of small-sample typing. Alternatively, one could use the minority alleles to estimate biopsy diversity, without attempting to reconstruct minority genotypes.

One potential use of diversity estimations would be as the basis for corrected mutational distances to be used in phylogeny inference. Simulations or heuristics could be used to establish the relationship between observed mutational distance between two biopsies, internal diversity of each biopsy, and the true mutational distance. Distances corrected according to this relationship could then be used in a distance-based phylogeny algorithm to produce trees whose branch lengths more accurately represented the underlying mutational frequencies. We are currently developing such an algorithm.

Accurate inference of branch lengths is important in distinguishing, for example, a mutation rate increase in a specific lineage (presumably due to a mutator mutation or epigenetic change) from a mutation rate increase at a given time across the entire tissue (presumably due to an environmental change, since it manifests in unrelated lineages). Naive tree-drawing based on uncorrected data, as shown by the results in this paper, cannot answer such questions as the branch lengths of its trees are not proportional to time. This is shown dramatically in real BE data, where no separation is seen between data collected from time points many years apart [1]. This situation makes it difficult to draw conclusions about changes in rate over time, though in some cases coherent patterns have been detected [1]. Genetic distances corrected for the bias inherent in bulk-biopsy sampling could allow much more accurate separation of neutral from non-neutral processes in the development of tissues and cancers.

**Fig 5:**
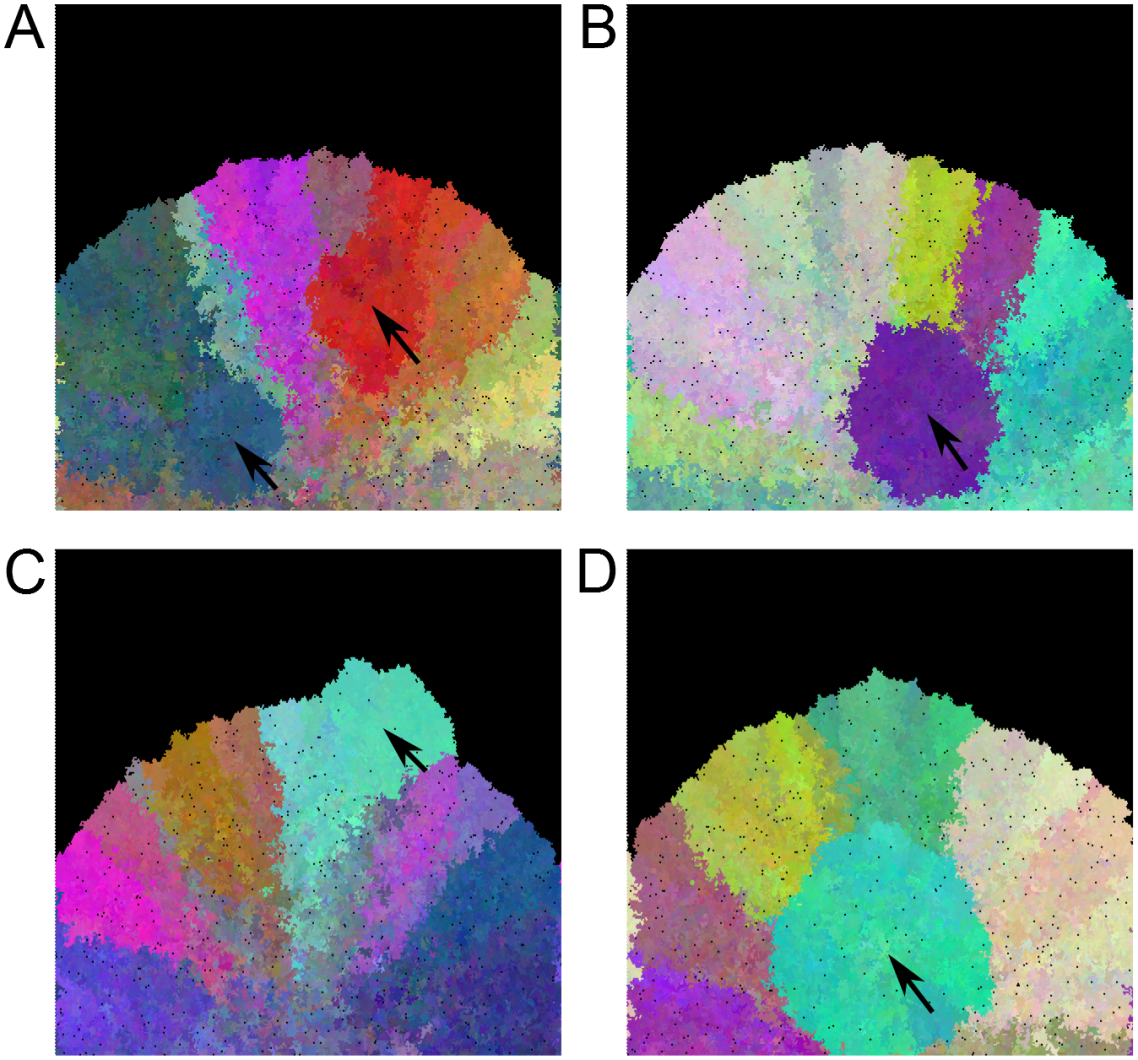
Simulation including a positively selected locus. Panels A-D represent the results of four independent simulations with selection. Black regions represent not-yet-colonized areas of the esophagus, and small black dots represent crypts which have died and not yet been replaced. Colors indicate the first three principal components of a PCA analysis of crypt genotypes at 100 neutral loci, essentially labeling distinct clones or cell lineages. Initial seeding of the esophagus was in the bottom-center of the figure. The left and right sides of the image wrap around. Arrows indicate clones carrying a selectively advantageous mutation. These images were chosen from a larger collection to illustrate typical selection signatures and do not represent a random sample. Selected clones appear more homogeneous and are round in shape whereas clones which expanded neutrally are wedge-shaped.

One further positive finding from this study is that the spread of a growing tissue tends to produce a characteristic fan-shaped pattern, as seen in Fig 1. As dense sampling of cancer and pre-cancer tissues becomes more feasible, it will become possible to detect this pattern or deviations from it which may indicate selection. An example is shown in Fig 5, which shows four typical results from a simulation with selected as well as neutral mutations. Note the disruption of the fan pattern by lateral growth of selected clones. The spatial distribution of clones within an expanding tumor or neoplasm may therefore reveal selective processes, as has been explored by [11] in colorectal cancer. In other words, these simulations provide predictions for the nascent field of tumor phylogeography. More work is needed both to detect these patterns and to assess their significance.

## Supporting Information

S1 Fig Rejection of the clock with 1000 neutral loci, *μ* = 0.001, equal allele frequencies. Heat-map showing the percentage of cases rejecting the molecular clock at the 5% level; lighter colors indicate lower rejection of the clock. These data correspond to S3 Table.
(TIFF)

S2 Fig Rejection of the clock with 1000 neutral loci, *μ* = 0.002, equal allele frequencies. Heat-map showing the percentage of cases rejecting the molecular clock at the 5% level; lighter colors indicate lower rejection of the clock. These data correspond to S4 Table.
(TIFF)

S3 Fig Rejection of the clock with 100 neutral loci, *μ* = 0.001, inferred allele frequencies. Heat-map showing the percentage of cases rejecting the molecular clock at the 5% level; lighter colors indicate lower rejection of the clock. These data correspond to S5 Table.
(TIFF)

S4 Fig Rejection of the clock with 100 neutral loci, *μ* = 0.001, equal allele frequencies. Heat-map showing the percentage of cases rejecting the molecular clock at the 5% level; lighter colors indicate lower rejection of the clock. These data correspond to S6 Table.
(TIFF)

S5 Fig Rejection of the clock with 100 neutral loci, *μ* = 0.002, equal allele frequencies. Heat-map showing the percentage of cases rejecting the molecular clock at the 5% level; lighter colors indicate lower rejection of the clock. These data correspond to S7 Table.
(TIFF)

S6 Fig Rejection of the clock with 100 neutral loci, *μ* = 0.004, equal allele frequencies. Heat-map showing the percentage of cases rejecting the molecular clock at the 5% level; lighter colors indicate lower rejection of the clock. These data correspond to S8 Table.
(TIFF)

S1 Table Rejection of the clock with 1000 neutral loci, *μ* = 0.001, inferred allele frequencies. *μ*, mutation rate per locus per generation. These data correspond to Fig 2 in the main manuscript.
(PDF)

S2 Table Rejection of the clock with 1000 neutral loci, *μ* = 0.002, inferred allele frequencies. *μ*, mutation rate per locus per generation. These data correspond to Fig 3 in the main manuscript.
(PDF)

S3 Table Rejection of the clock with 1000 neutral loci, *μ* = 0.001, equal allele frequencies. *μ*, mutation rate per locus per generation. These data correspond to S1 Fig.
(PDF)

S4 Table Rejection of the clock with 1000 neutral loci, *μ* = 0.002, equal allele frequencies. *μ*, mutation rate per locus per generation. These data correspond to S2 Fig.
(PDF)

S5 Table Rejection of the clock with 100 neutral loci, *μ* = 0.001, inferred allele frequencies. *μ*, mutation rate per locus per generation. These data correspond to S3 Fig. A small proportion of runs could not be completed due to too many invariant biopsies, leading to sample sizes less than 500. For the last three entries in cutoff 90%, sample sizes were 499, 498, 497. For the last six entries in cutoff 100%, sample sizes were 497, 496, 494, 492, 492, 487.
(PDF)

S6 Table Rejection of the clock with 100 neutral loci, *μ* = 0.001, equal allele frequencies. *μ*, mutation rate per locus per generation. These data correspond to S4 Fig. A small proportion of runs could not be completed due to too many invariant biopsies, leading to sample sizes less than 500. For the last three entries in cutoff 90%, sample sizes were 499, 498, 497. For the last six entries in cutoff 100%, sample sizes were 497, 496, 494, 492, 492, 487.
(PDF)

S7 Table Rejection of the clock with 100 neutral loci, *μ* = 0.002, equal allele frequencies. *μ*, mutation rate per locus per generation. These data correspond to S5 Fig.
(PDF)

S8 Table Rejection of the clock with 100 neutral loci, *μ* = 0.004, equal allele frequencies. *μ*, mutation rate per locus per generation. These data correspond to S6 Fig.
(PDF)
