# Supplementary figures and images for: Bulk Genotyping of Biopsies Can Create Spurious Evidence for Hetereogeneity in Mutation Content
Source: PLoS Comput Biol. 2016 Apr 22;12(4):e1004413. doi: 10.1371/journal.pcbi.1004413 (PMC4841575; doi:10.1371/journal.pcbi.1004413)

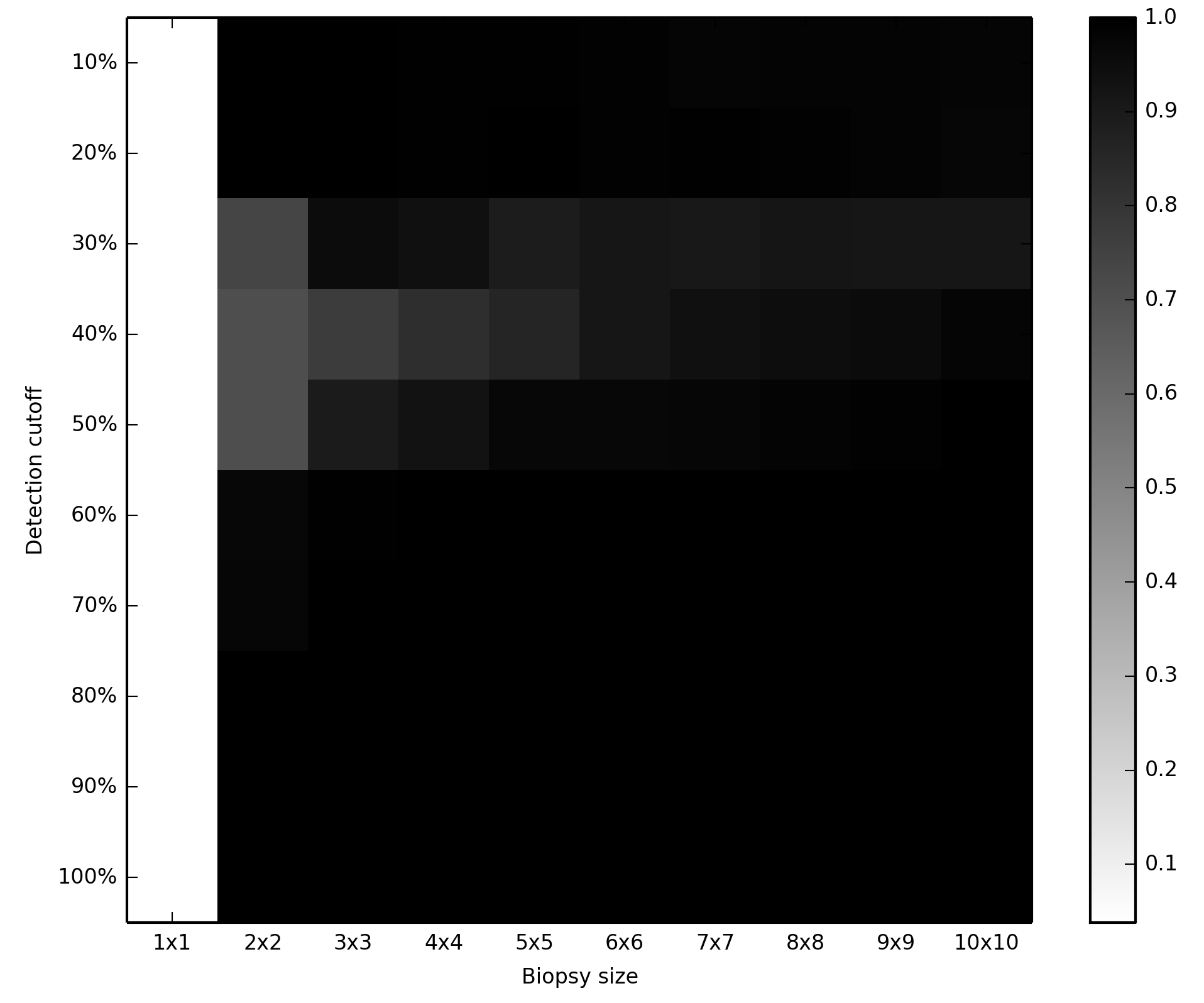

Supplement: S1 Fig — Heat-map showing the percentage of cases rejecting the molecular clock at the 5% level; lighter colors indicate lower rejection of the clock. These data correspond to S3 Table. (TIFF) [file pcbi.1004413.s001.tiff]

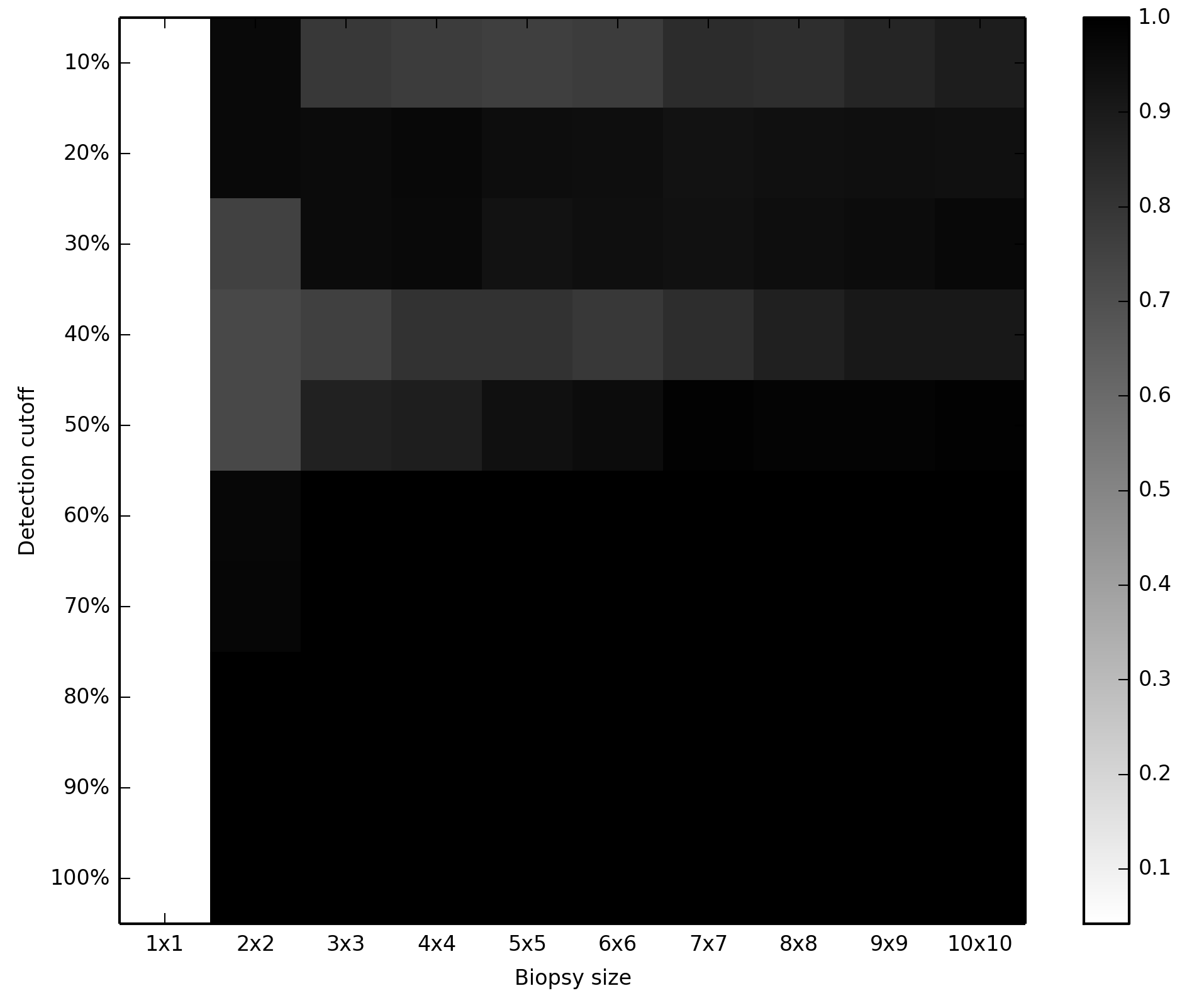

Supplement: S2 Fig — Heat-map showing the percentage of cases rejecting the molecular clock at the 5% level; lighter colors indicate lower rejection of the clock. These data correspond to S4 Table. (TIFF) [file pcbi.1004413.s002.tiff]

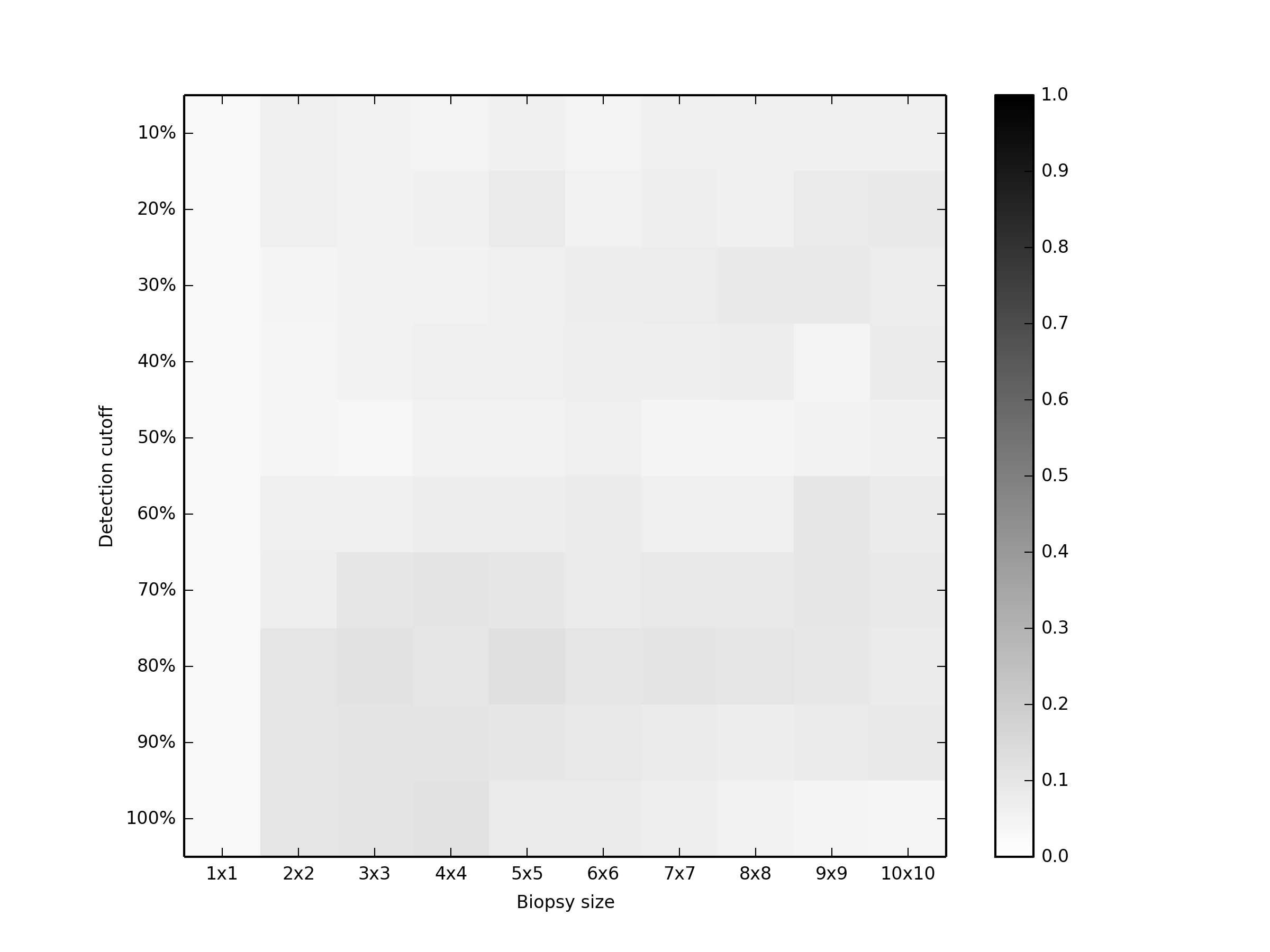

Supplement: S3 Fig — Heat-map showing the percentage of cases rejecting the molecular clock at the 5% level; lighter colors indicate lower rejection of the clock. These data correspond to S5 Table. (TIFF) [file pcbi.1004413.s003.tiff]

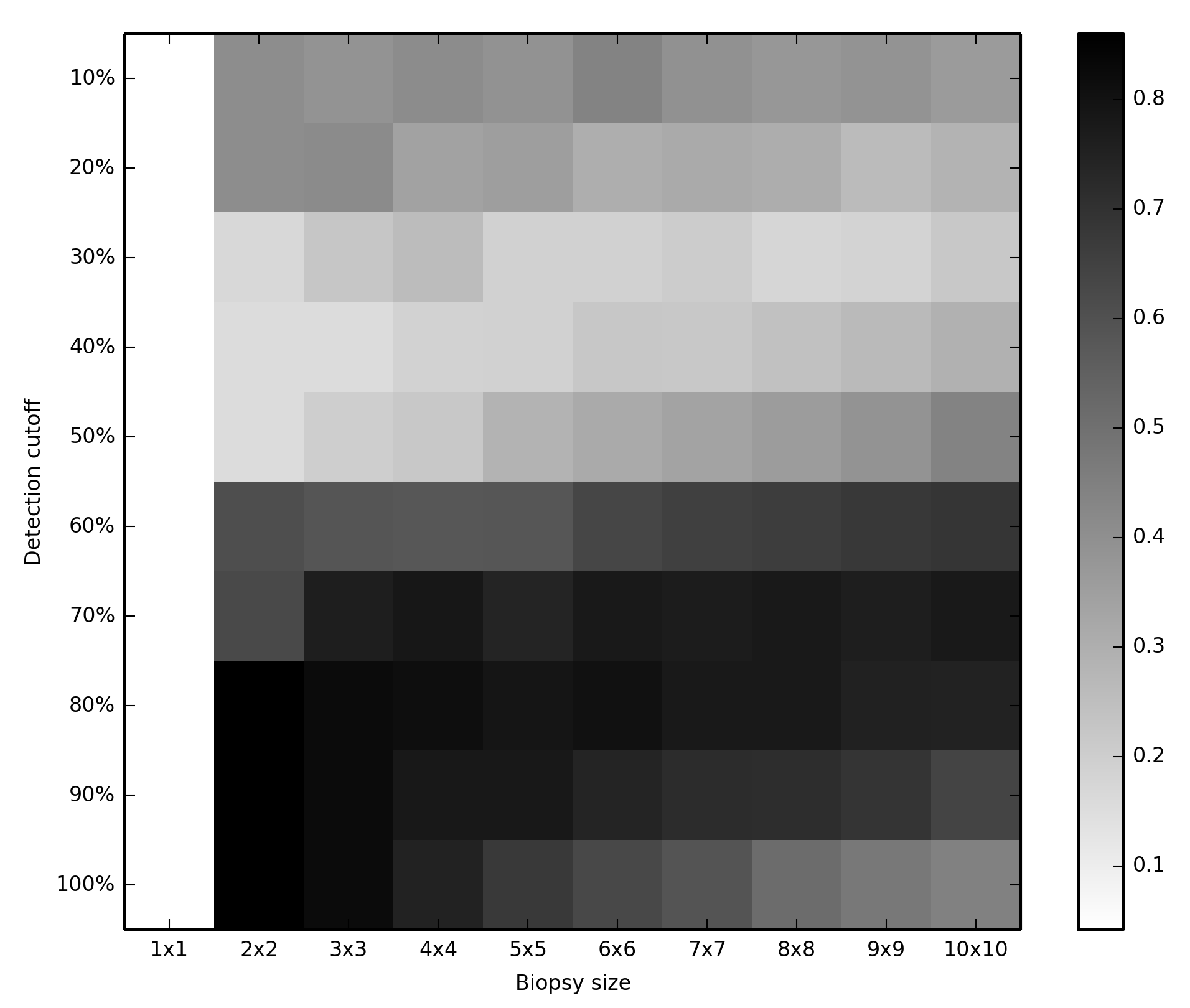

Supplement: S4 Fig — Heat-map showing the percentage of cases rejecting the molecular clock at the 5% level; lighter colors indicate lower rejection of the clock. These data correspond to S6 Table. (TIFF) [file pcbi.1004413.s004.tiff]

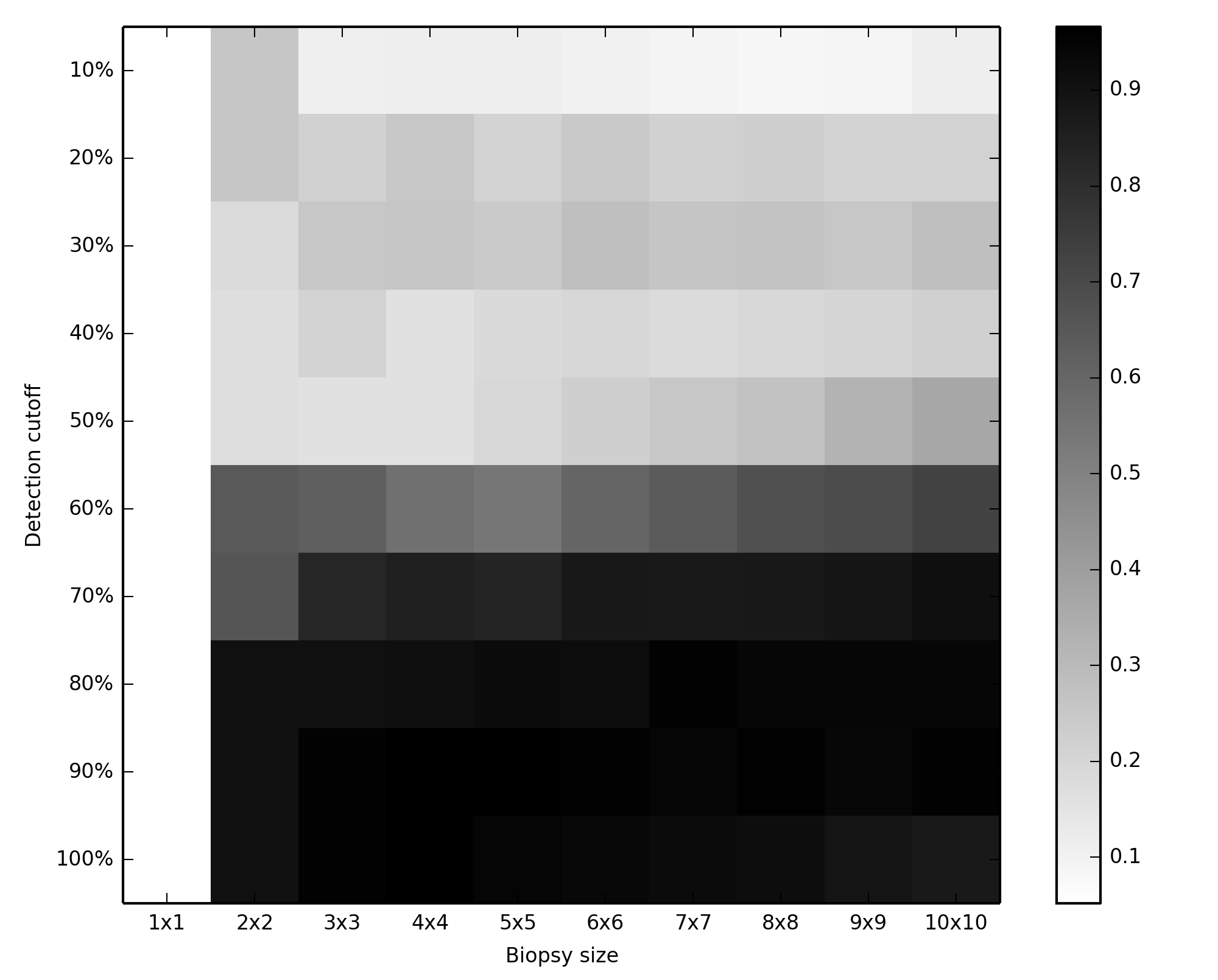

Supplement: S5 Fig — Heat-map showing the percentage of cases rejecting the molecular clock at the 5% level; lighter colors indicate lower rejection of the clock. These data correspond to S7 Table. (TIFF) [file pcbi.1004413.s005.tiff]

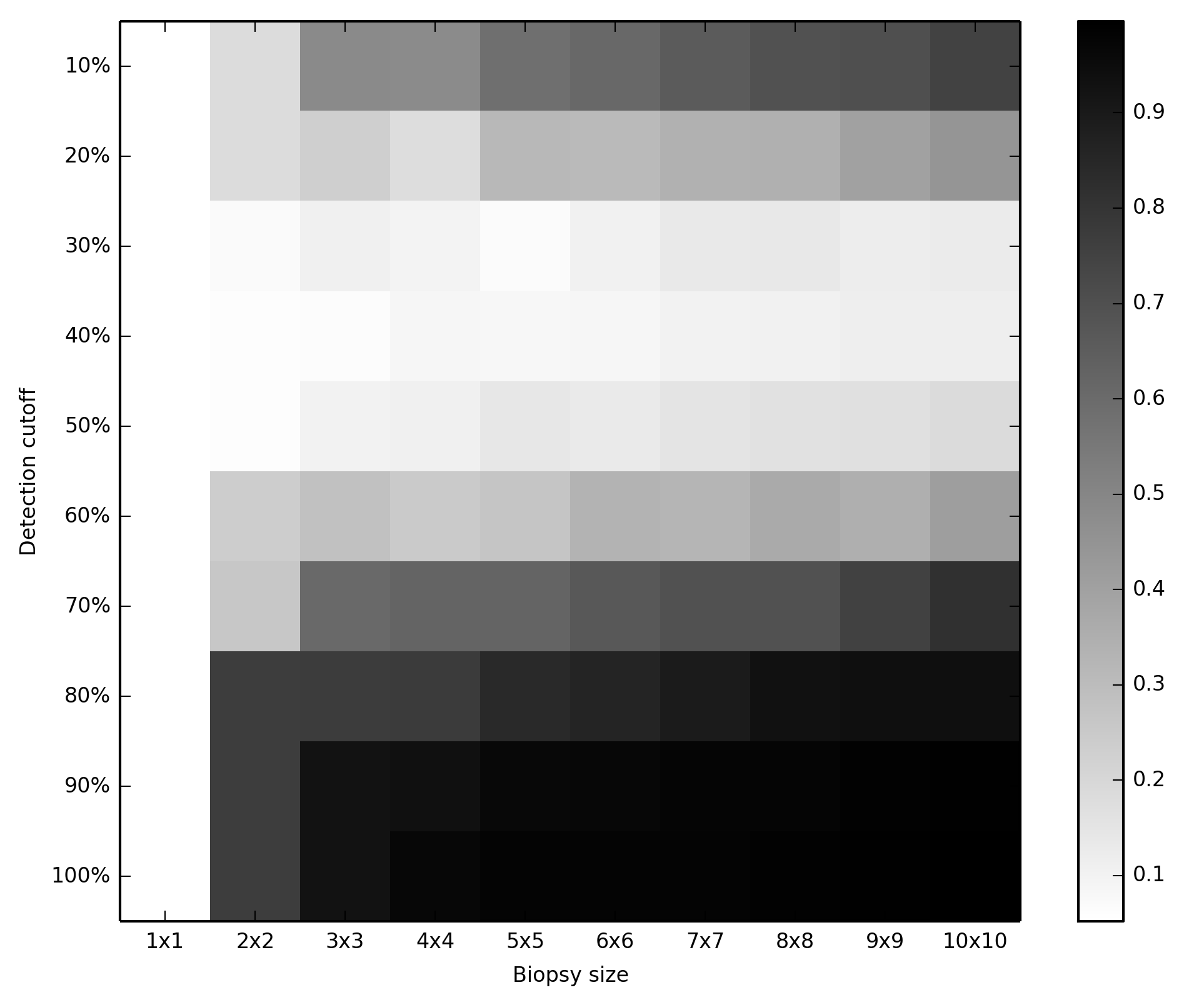

Supplement: S6 Fig — Heat-map showing the percentage of cases rejecting the molecular clock at the 5% level; lighter colors indicate lower rejection of the clock. These data correspond to S8 Table. (TIFF) [file pcbi.1004413.s006.tiff]
