# Supplementary material for: Bulk Genotyping of Biopsies Can Create Spurious Evidence for Hetereogeneity in Mutation Content
Source: PLoS Comput Biol. 2016 Apr 22;12(4):e1004413. doi: 10.1371/journal.pcbi.1004413 (PMC4841575; doi:10.1371/journal.pcbi.1004413)
Supplement: S1 Table — μ, mutation rate per locus per generation. These data correspond to Fig 2 in the main manuscript. (PDF) [file pcbi.1004413.s007.pdf]

Table S1. Rejection of the clock with 1000 neutral loci, $\mu = 0.001$, inferred allele frequencies

	Biopsy size									
Cutoff	1x1	2x2	3x3	4x4	5x5	6x6	7x7	8x8	9x9	10x10
10	0.066	0.536	0.602	0.598	0.610	0.586	0.592	0.644	0.680	0.714
20	0.066	0.536	0.522	0.516	0.528	0.536	0.522	0.548	0.584	0.566
30	0.066	0.234	0.370	0.394	0.374	0.432	0.426	0.468	0.498	0.538
40	0.064	0.234	0.262	0.326	0.376	0.408	0.478	0.494	0.528	0.570
50	0.066	0.234	0.302	0.344	0.452	0.464	0.530	0.568	0.602	0.638
60	0.066	0.604	0.640	0.656	0.700	0.738	0.788	0.794	0.824	0.838
70	0.066	0.638	0.818	0.856	0.848	0.902	0.902	0.902	0.928	0.930
80	0.066	0.934	0.914	0.890	0.924	0.936	0.940	0.940	0.936	0.952
90	0.066	0.934	0.960	0.950	0.954	0.966	0.958	0.956	0.952	0.964
100	0.066	0.934	0.960	0.954	0.950	0.948	0.944	0.954	0.948	0.938

μ, mutation rate per locus per generation
These data correspond to Fig. 2 in the main manuscript.
