# Supplementary material for: Bulk Genotyping of Biopsies Can Create Spurious Evidence for Hetereogeneity in Mutation Content
Source: PLoS Comput Biol. 2016 Apr 22;12(4):e1004413. doi: 10.1371/journal.pcbi.1004413 (PMC4841575; doi:10.1371/journal.pcbi.1004413)
Supplement: S2 Table — μ, mutation rate per locus per generation. These data correspond to Fig 3 in the main manuscript. (PDF) [file pcbi.1004413.s008.pdf]

Table S2. Rejection of the clock with 1000 neutral loci, $\mu = 0.002$, inferred allele frequencies

	Biopsy size									
Cutoff	1x1	2x2	3x3	4x4	5x5	6x6	7x7	8x8	9x9	10x10
10	0.120	0.676	0.694	0.674	0.704	0.726	0.712	0.718	0.738	0.732
20	0.120	0.676	0.63	0.646	0.676	0.678	0.664	0.684	0.722	0.750
30	0.120	0.338	0.502	0.574	0.538	0.554	0.544	0.572	0.582	0.628
40	0.120	0.302	0.340	0.424	0.418	0.470	0.486	0.544	0.622	0.642
50	0.120	0.302	0.300	0.386	0.454	0.476	0.558	0.604	0.678	0.700
60	0.120	0.588	0.666	0.680	0.708	0.748	0.802	0.830	0.868	0.892
70	0.120	0.618	0.822	0.882	0.866	0.910	0.938	0.944	0.960	0.972
80	0.120	0.924	0.930	0.922	0.956	0.964	0.970	0.988	0.982	0.974
90	0.120	0.924	0.976	0.980	0.986	0.988	0.992	0.990	0.992	0.996
100	0.120	0.926	0.976	0.988	0.994	0.996	0.998	0.998	0.990	0.988

μ, mutation rate per locus per generation
These data correspond to Fig. 3 in the main manuscript.
