# Supplementary material for: Bulk Genotyping of Biopsies Can Create Spurious Evidence for Hetereogeneity in Mutation Content
Source: PLoS Comput Biol. 2016 Apr 22;12(4):e1004413. doi: 10.1371/journal.pcbi.1004413 (PMC4841575; doi:10.1371/journal.pcbi.1004413)
Supplement: S4 Table — μ, mutation rate per locus per generation. These data correspond to S2 Fig. (PDF) [file pcbi.1004413.s010.pdf]

Table S4. Rejection of the clock with 1000 neutral loci, $\mu = 0.002$, equal allele frequencies

	Biopsy size									
Cutoff	1x1	2x2	3x3	4x4	5x5	6x6	7x7	8x8	9x9	10x10
10	0.042	0.962	0.790	0.774	0.762	0.774	0.832	0.826	0.860	0.890
20	0.042	0.962	0.958	0.968	0.948	0.946	0.928	0.936	0.942	0.934
30	0.042	0.756	0.958	0.960	0.926	0.942	0.930	0.944	0.952	0.964
40	0.042	0.730	0.758	0.808	0.806	0.790	0.828	0.880	0.910	0.906
50	0.042	0.730	0.870	0.886	0.940	0.954	0.986	0.984	0.984	0.988
60	0.042	0.972	0.998	0.994	0.998	0.998	1.000	1.000	1.000	1.000
70	0.042	0.974	1.000	1.000	1.000	1.000	1.000	1.000	1.000	1.000
80	0.042	1.000	1.000	1.000	1.000	1.000	1.000	1.000	1.000	1.000
90	0.042	1.000	1.000	1.000	1.000	1.000	1.000	1.000	1.000	1.000
100	0.042	1.000	1.000	1.000	1.000	1.000	1.000	1.000	1.000	1.000

μ, mutation rate per locus per generation
These data correspond to Supporting Figure S2.
