# Supplementary material for: Bulk Genotyping of Biopsies Can Create Spurious Evidence for Hetereogeneity in Mutation Content
Source: PLoS Comput Biol. 2016 Apr 22;12(4):e1004413. doi: 10.1371/journal.pcbi.1004413 (PMC4841575; doi:10.1371/journal.pcbi.1004413)
Supplement: S6 Table — μ, mutation rate per locus per generation. These data correspond to S4 Fig. A small proportion of runs could not be completed due to too many invariant biopsies, leading to sample sizes less than 500. For the last three entries in cutoff 90%, sample sizes were 499, 498, 497. For the last six entries in cutoff 100%, sample sizes were 497, 496, 494, 492, 492, 487. (PDF) [file pcbi.1004413.s012.pdf]

Table S6. Rejection of the clock with 100 neutral loci, $\mu = 0.001$, equal allele frequencies

	Biopsy size									
Cutoff	1x1	2x2	3x3	4x4	5x5	6x6	7x7	8x8	9x9	10x10
10	0.042	0.408	0.384	0.410	0.392	0.436	0.394	0.376	0.386	0.364
20	0.042	0.408	0.414	0.342	0.352	0.304	0.316	0.306	0.262	0.282
30	0.042	0.168	0.226	0.258	0.192	0.192	0.208	0.176	0.182	0.218
40	0.042	0.154	0.156	0.188	0.190	0.224	0.218	0.242	0.264	0.292
50	0.042	0.154	0.200	0.220	0.288	0.314	0.338	0.360	0.390	0.436
60	0.042	0.610	0.588	0.580	0.584	0.634	0.654	0.662	0.676	0.690
70	0.042	0.624	0.764	0.786	0.744	0.774	0.768	0.776	0.762	0.776
80	0.042	0.860	0.824	0.814	0.790	0.800	0.776	0.774	0.750	0.746
90	0.042	0.860	0.822	0.782	0.778	0.742	0.718	0.715	0.693	0.642
100	0.042	0.860	0.822	0.746	0.672	0.627	0.591	0.512	0.476	0.448

μ, mutation rate per locus per generation
These data correspond to Supporting Figure S4.
A small proportion of runs could not be completed due to too many invariant biopsies, leading to sample sizes less than 500. For the last three entries in cutoff 90%, sample sizes were 499, 498, 497. For the last six entries in cutoff 100%, sample sizes were 497, 496, 494, 492, 492, 487.
