# Supplementary material for: Bulk Genotyping of Biopsies Can Create Spurious Evidence for Hetereogeneity in Mutation Content
Source: PLoS Comput Biol. 2016 Apr 22;12(4):e1004413. doi: 10.1371/journal.pcbi.1004413 (PMC4841575; doi:10.1371/journal.pcbi.1004413)
Supplement: S7 Table — μ, mutation rate per locus per generation. These data correspond to S5 Fig. (PDF) [file pcbi.1004413.s013.pdf]

Table S7. Rejection of the clock with 100 neutral loci, $\mu = 0.002$, equal allele frequencies

	Biopsy size									
Cutoff	1x1	2x2	3x3	4x4	5x5	6x6	7x7	8x8	9x9	10x10
10	0.052	0.258	0.110	0.116	0.114	0.104	0.092	0.082	0.088	0.116
20	0.052	0.258	0.218	0.252	0.212	0.248	0.218	0.224	0.208	0.216
30	0.052	0.184	0.254	0.258	0.242	0.284	0.260	0.266	0.252	0.282
40	0.052	0.172	0.206	0.162	0.186	0.196	0.184	0.192	0.204	0.222
50	0.052	0.172	0.162	0.162	0.192	0.224	0.254	0.272	0.324	0.368
60	0.052	0.646	0.626	0.564	0.544	0.604	0.640	0.678	0.692	0.726
70	0.052	0.662	0.830	0.854	0.834	0.876	0.870	0.878	0.890	0.912
80	0.052	0.902	0.908	0.910	0.924	0.922	0.954	0.944	0.944	0.944
90	0.052	0.902	0.954	0.960	0.960	0.956	0.948	0.958	0.940	0.954
100	0.052	0.902	0.954	0.966	0.948	0.932	0.926	0.918	0.894	0.870

μ, mutation rate per locus per generation
These data correspond to Supporting Figure S5.
