# Supplementary material for: Bulk Genotyping of Biopsies Can Create Spurious Evidence for Hetereogeneity in Mutation Content
Source: PLoS Comput Biol. 2016 Apr 22;12(4):e1004413. doi: 10.1371/journal.pcbi.1004413 (PMC4841575; doi:10.1371/journal.pcbi.1004413)
Supplement: S8 Table — μ, mutation rate per locus per generation. These data correspond to S6 Fig. (PDF) [file pcbi.1004413.s014.pdf]

Table S8. Rejection of the clock with 100 neutral loci, $\mu = 0.004$, equal allele frequencies

	Biopsy size									
Cutoff	1x1	2x2	3x3	4x4	5x5	6x6	7x7	8x8	9x9	10x10
10	0.052	0.184	0.484	0.480	0.586	0.610	0.658	0.696	0.702	0.748
20	0.052	0.184	0.230	0.180	0.314	0.308	0.342	0.344	0.402	0.444
30	0.052	0.072	0.110	0.098	0.068	0.104	0.134	0.140	0.122	0.128
40	0.052	0.060	0.066	0.088	0.082	0.088	0.100	0.104	0.116	0.116
50	0.052	0.060	0.102	0.110	0.144	0.132	0.154	0.166	0.168	0.186
60	0.052	0.240	0.282	0.250	0.266	0.332	0.328	0.366	0.350	0.410
70	0.052	0.262	0.604	0.626	0.624	0.670	0.694	0.696	0.754	0.814
80	0.052	0.768	0.774	0.778	0.838	0.860	0.896	0.928	0.940	0.938
90	0.052	0.768	0.924	0.934	0.960	0.970	0.974	0.976	0.982	0.986
100	0.052	0.768	0.924	0.968	0.980	0.978	0.980	0.984	0.986	0.996

μ, mutation rate per locus per generation
These data correspond to Supporting Figure S6.
